# Complete Genome Sequence of a Jumbo Bacteriophage, Escherichia Phage vB_EcoM_EC001

**DOI:** 10.1128/mra.00017-22

**Published:** 2022-02-03

**Authors:** Stevan Cucić, Andrew M. Kropinski, Janet Lin, Cezar M. Khursigara, Hany Anany

**Affiliations:** a Guelph Research and Development Centre, Agriculture and AgriFood Canada, Guelph, Ontario, Canada; b Department of Molecular and Cellular Biology, College of Biological Science, University of Guelph, Guelph, Ontario, Canada; c Department of Food Science, Ontario Agricultural College, University of Guelph, Guelph, Ontario, Canada; d Department of Pathobiology, Ontario Agricultural College, University of Guelph, Guelph, Ontario, Canada; Portland State University

## Abstract

Here, we report the genome sequence of a jumbo Escherichia phage vB_EcoM_EC001, a myovirus isolated from primary sludge using enterohemorrhagic Escherichia coli O157:H7. The genome is 240,200 bp long and has 270 predicted coding sequences, including a tryptophanyl tRNA gene. It belongs to genus *Seoulvirus*.

## ANNOUNCEMENT

Enterohemorrhagic Escherichia coli (EHEC) can cause severe disease, including hemolytic uremic syndrome (1). Phages infecting EHEC strains are of interest due to their potential utility as biocontrol, therapeutic, and diagnostic agents (2, 3).

Standard approaches to phage isolation are biased toward phages that produce clearly visible plaques. The diversity of coliphages producing clearly visible plaques has been probed extensively. We sought to bias our phage isolation toward large genome size phages by using 0.3% agar instead of the standard 0.4% to 0.7% and selecting pinprick plaques. Pinprick plaques on lower agar concentration overlay can indicate larger phage particles and consequently larger genome sizes (4). Large genome phages encode a preponderance of hypothetical proteins without biochemically characterized homologs and therefore merit investigation (5).

EC001 was isolated in 2017 by enriching the unfiltered supernatant of primary sludge from the Guelph, Ontario, wastewater treatment plant with a cocktail of Shiga toxin-producing E. coli (STEC) strains (O157, O111, O26, O103, O145, O121, and O45, with respective strain designations ATCC 700927, HA2018015, HA2018016, HA2018017, HA2018018, HA2018019, and HA2018020), adding 5× of tryptic soy broth (TSB) to the wastewater sample, and incubating the sample for 24 hours at 25°C. Thereafter, a few milliliters of the enriched sample were filtered through 0.45-μm cellulose acetate syringe filters and spotted on 0.3% agar semisolid TSB (ssTSB) containing E. coli O157:H7. A pinprick plaque was passaged three times and propagated using the soft agar method and incubation at 25°C (6).

The PureLink viral DNA/RNA minikit (Thermo Fisher Scientific) was used to extract phage genomic DNA from the crude phage lysate according to the supplier’s instructions. DNA elution was done with molecular biology-grade water. DNA was normalized to 200 ng as quantified by fluorimetry (Qubit, Thermo Fisher Scientific) and mechanically sheared using an M220 instrument (Covaris). The next-generation sequencing (NGS) library was prepared using the NxSeq AmpFREE low DNA library kit (Lucigen) sequenced with the MiSeq sequencer (Illumina) using a 2 × 300-bp MiSeq reagent kit v3 (600 cycles) (Illumina) with an average coverage depth of 221-fold. All kits were used according to the manufacturer’s protocols. The genome was *de novo* assembled using the SeqManNGen15 (DNAStar) using a base of 200,000 reads (out of ∼1.8 million reads) and the approximate genome size observed from pulsed-field gel electrophoresis (∼240 Kbp). The draft genome was realigned to that of Salmonella phage JN03 (GenBank accession number MT799840) using progressiveMauve (7). A circular contig of 240,200 bp was obtained. Genome annotation was performed using RAST and MetaGeneAnnotator (8, 9) with protein names verified using Pfam (10). All tools were run with default parameters.

**FIG 1:**
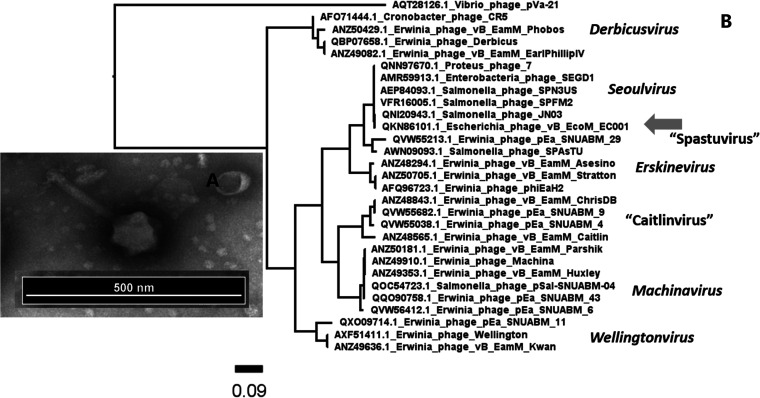
(A) Uranyl acetate transmission electron micrograph of vB_EcoM_EC001 (phage sample was prepared for imaging as in reference 12). (B) Phylogenetic tree generated using phylogeny.fr default settings coupled with FigTree based on the amino acid sequence of large subunit terminase protein. The arrow is pointing to the vB_EcoM_EC001 location in the tree. All selected phages were obtained from ICTV Master Species List 36 (https://talk.ictvonline.org/files/master-species-lists/m/msl/12314).

EC001 has icosahedral capsids approximately 120 nm in diameter and tails 190 nm long (Fig. 1A). It has a 240.2-kbp linear, circularly permuted double-stranded DNA (dsDNA) genome with a GC content of 48.5%. Of the 269 predicted protein-coding sequences, only 59 have predicted functions. A phylogenetic tree made using phylogeny.fr (11) based on the amino acid sequence of its terminase large subunit indicates that its closest relative is Salmonella phage JN03 (GenBank accession number MT799840.1) (Fig. 1B), a member of the *Seoulvirus* genus.

### Data availability.

The complete genome sequence of Escherichia phage EC001 is accessible at GenBank using the accession number MN445185.1. The raw sequence reads are available at NCBI SRA database with accession number SRP351973.
